# Antioxidant Property of Aerial Parts and Root of *Phyllanthus fraternus* Webster, an Important Medicinal Plant

**DOI:** 10.1155/2014/692392

**Published:** 2014-01-23

**Authors:** Richa Upadhyay, Jitendra Kumar Chaurasia, Kavindra Nath Tiwari, Karuna Singh

**Affiliations:** ^1^Department of Botany, MMV, Banaras Hindu University, Varanasi, Uttar Pradesh 221005, India; ^2^Department of Applied Science, Kashi Institute of Technology, Varanasi, Uttar Pradesh 221307, India; ^3^Department of Zoology, MMV, Banaras Hindu University, Varanasi, Uttar Pradesh 221005, India

## Abstract

In present study free radical scavenging potential of aerial parts and root of *Phyllanthus fraternus* was investigated. Extraction was done in water and ethanol. Total antioxidant capacity was measured by DPPH free radical scavenging method; ethanolic extract of aerial part was most potent in activity with 50% inhibition at 258 **μ**g/mL concentration. Lipid peroxidation (LPO) was measured in terms of thiobarbituric acid-reactive substances (TBARS) by using egg-yolk homogenates as lipid-rich media with EC_50_ of aerial part (ethanolic) 1522 **μ**g/mL which was found to be most active. Superoxide (SO) radical scavenging activity was measured using riboflavin-light-nitroblue tetrazolium assay. Ethanolic and aqueous extract of both aerial part and root was almost similar in superoxide radical scavenging activity. Reducing power was determined on the basis of Fe^3+^-Fe^2+^ transformation in the presence of extract. Total phenolic and flavonoid contents were also measured by spectroscopic method. Results showed that the ethanolic fraction of aerial part is most active towards antioxidant potential and this activity is related to its polyphenolic content and reducing potential. Thus, *P. fraternus* extract can be used as potent natural antioxidant.

## 1. Introduction

Natural antioxidants have generated considerable interest in preventive medicine. Free radicals play very crucial role in occurrence of various neurodegenerative diseases and also accelerate aging [1, 2]. Free radicals associated with oxygen known as reactive oxygen species (ROS) are continuously produced during normal physiological processes. There are so many scavengers such as glutathione (GSH), super oxide dismutase (SOD), catalase (CAT), and so forth present in body for balancing of free radicals. In pathological conditions free radical formation is very high and oxidative stress cause damage of DNA, protein, lipid, and so forth. In this condition, internal antioxidants become insufficient for balancing of free radicals so external antioxidants are needed to prevent the oxidative damages by directly reacting with ROS, quenching them and/or chelating catalytic metal ions and also by scavenging free oxygen [3]. Plants contain a wide variety of free radical scavenging molecules, such as flavonoids, anthocyanins, carotenoids, dietary glutathione, vitamins, and endogenous metabolites [4, 5]. Among the various natural antioxidants, phenolic compounds possess significant antioxidant potential because it has characteristic of quenching oxygen-derived free radicals by donating a hydrogen atom or an electron to the free radicals [6].

*Phyllanthus fraternus* is a medicinal herb widely distributed in most tropical and subtropical countries [7]. It is extensively used in folk medicine in India and most other countries in the treatment of a broad spectrum of diseases, such as disturbances of the kidney and urinary bladder, intestinal infections, diabetes, and hepatitis for thousands of years [8, 9]. It also possesses significant astringent, deobstruent, diuretic, and antiseptic activity. There is no extensive study regarding antioxidant activity of different plant parts of *P. fraternus*. Koffuor and Amoateng [10] have reported antioxidant and anticoagulant property of fruit parts of *P. fraternus*. So, we have studied the antioxidant activity of ethanol and aqueous extracts derived from its aerial part and root.

## 2. Materials and Methods

### 2.1. Chemicals

1,1-Diphenyl,2-picryl hydrazyl (DPPH), nitroblue tetrazolium (NBT), riboflavin, L-methionine, thiobarbituric acid (TBA), ethylenediaminetetraacetic acid (EDTA), ascorbic acid, gallic acid, rutin and trichloroacetic acid (TCA), potassium ferricyanide (“K_3_[Fe(CN)_6_]”), and ferric chloride (“FeCl_3_”) were purchased from Hi-Media Ltd. All reagents were of analytical grade.

### 2.2. Plant Collection and Extract Preparation

Plants of *P. fraternus* were collected from the campus of Banaras Hindu University, Varanasi, during months of August and September. The plant was taxonomically identified by Professor N. K. Dubey, Botany Department, Banaras Hindu University. For preparation of extract, aerial parts and roots were washed thoroughly under running tap water, oven dried at 50–60°C for two days, and then powdered in a mechanical grinder. For aqueous extract preparation, about 250 g of the powdered material was boiled in 500 mL distilled water for 30 minutes, kept for 3 days with intermittent shaking [11]. For preparation of ethanolic extract each powdered sample (100 g) was extracted with 250 mL of ethanol by using a soxhlet extractor [12]. Extract was then filtered and evaporated to dryness at a 45°C with rotary evaporator. Their % yield (w/w) was calculated with original amount of coarse powder used for extraction. It was about 20.89 and 33% for ethanolic and aqueous aerial part, respectively. For root extract % yield was 10.56 and 13% for ethanolic and aqueous fraction, respectively.

### 2.3. DPPH Radical Scavenging Activity

The free radical scavenging activity of the extracts, based on the scavenging activity of the stable 1, 1-diphenyl-2-picrylhydrazyl (DPPH) free radical, was determined by slightly modified method described by Brand-Williams et al. [13]. Different concentration of plant extract were added to 3 mL of a 0.004% methanolic solution of DPPH and incubated for 15 minutes at room temperature. Absorbance was recorded at 517 nm by using spectrophotometer (Thermo Scientific UV1).

### 2.4. Lipid Peroxidation Assay

A modified thiobarbituric acid-reactive species (TBARS) assay [14] was used to measure the lipid peroxide formed, using egg-yolk homogenates as lipid-rich media [15]. Malondialdehyde (MDA), a secondary product of the oxidation of polyunsaturated fatty acids, reacts with two molecules of thiobarbituric acid (TBA), yielding a pinkish red chromogen with an absorbance maximum at 532 nm [16]. Egg homogenate (250 *μ*L, 10% in distilled water, v/v) and 50 *μ*L of extract were mixed in a test tube and the volume was made up to 500 *μ*L, by adding distilled water. Finally, 25 *μ*L “FeSO_4_” (0.07 M) was added to the above mixture and incubated for 30 min, to induce lipid peroxidation. Thereafter, 750 *μ*L of 20% acetic acid (pH 3.5) and 750 *μ*L of 0.8% TBA (w/v) (prepared in 1.1% sodium dodecyl sulphate) and 25 *μ*L 20% TCA were added, vortexed, and then heated in a boiling water bath for 60 min. After cooling, 3.0 mL of 1-butanol was added to each tube and centrifuged at 3000 rpm for 10 min. The absorbance of the organic upper layer was measured against 3 mL butanol at 532 nm. For the blank 50 *μ*L of distilled water was used in place of the extract.

### 2.5. Superoxide Radical Scavenging Property

This assay was based on the capacity of the extract to inhibit the photochemical reduction of nitroblue tetrazolium (NBT) [17]. In brief, each 3 mL reaction mixture contained 0.01 M phosphate buffer solution (PBS) (pH 7.8), 130 mM methionine, 60 *μ*M riboflavin, 0.5 mM EDTA, 0.75 mM NBT, and 0.5 mL of test sample solution. It was kept in front of fluorescent light for 6 minutes and absorbance was taken at 560 nm. Identical tubes containing reaction mixture were kept in the dark and served as controls. The percentage inhibition of superoxide generation was measured by comparing the absorbance of the control and those of the reaction mixture containing test sample. The blank was 0.01 M PBS.

### 2.6. Reducing Power (RP)

The reducing power of both aqueous and ethanolic extracts of *P. fraternus* was determined according to the method described by [18]. Different concentrations (50–400 *μ*g) of extracts were mixed with phosphate buffer (2.5 mL, 0.2 M, pH 6.6) and potassium ferricyanide [“K_3_Fe(CN)_6_”] (2.5 mL, 1%). The mixture was incubated at 37°C for 20 min after that 2.5 mL of trichloroacetic acid (TCA, 10%) was added to the mixture which was then centrifuged at 1000 rpm for 10 min. The upper organic layer of solution (2.5 mL) was taken and mixed with distilled water (2.5 mL) and “FeCl_3_” (0.5 mL, 0.1%), and the absorbance of reaction mixture was measured at 700 nm. Increased absorbance of the reaction mixture indicated high reducing power. Ascorbic acid was used as standard.

### 2.7. Measurement of Total Phenolics (TP)

TP concentration was measured by Folin-Ciocalteu assay [19]. Briefly, 1 mL of distilled water, 0.1 mL of 1 mg/mL sample, and 0.2 mL of Folin-Ciocalteu reagent were added in test tube; then contents were mixed and allowed to stand for 5–8 min at room temperature. Next, 2 mL of 7% sodium carbonate solution were added, followed by 0.7 mL distilled water to make 3 mL reaction mixture. Solutions were mixed and allowed to stand at room temperature for 15 min, and then absorbance was recorded at 750 nm. Phenolic contents were estimated by using a standard curve obtained from various concentration of gallic acid and expressed as milligrams per gram of gallic acid equivalents (GAE).

### 2.8. Measurement of Total Flavonoids (TF)

AlCl_3_ colorimetric method was used for TF determination [20]. Each plant extract (0.1 mL of 10 mg/mL) in ethanol was mixed with 0.1 mL of 2% AlCl_3_, 0.1 mL of 1 M potassium acetate, and 2.7 mL of ethanol. The reaction mixture was kept at room temperature for 30 min and absorbance was taken at 415 nm. TF content was calculated using rutin as standard and expressed as milligrams per gram of rutin equivalents (RE).

### 2.9. Determination of Percentage Inhibition and Statistical Analysis

Percentage inhibition was calculated as [*A*_0_ − *A*_*t*_/*A*_0_ × 100].  *A*_0_ is the absorbance of the control and *A*_*t*_ is the absorbance of the test samples/standard. All experiments were performed in triplicate and data are expressed as means ± SE. Statistical comparisons were made by means of one-way ANOVA test followed by post hoc analysis with Dunnett test by using SPSS (version 16). *P* values ≤0.001 were considered highly significant. EC_50_ values were calculated from linear regression analysis.

## 3. Results 

### 3.1. DPPH Radical Scavenging Assay

From Table 1 it is evident that all extracts have significant free radical scavenging activity. Ethanolic extract of aerial part have greater scavenging activity (EC_50_ = 258 *μ*g/mL) than aqueous extract (EC_50_ = 360 *μ*g/mL). Similarly, ethanolic extracts of roots also have greater activity (EC_50_ = 337 *μ*g/mL) than its aqueous extract (EC_50_ = 3038 *μ*g/mL).

### 3.2. Superoxide Scavenging Assay

Both the aqueous and ethanolic extract of aerial part exhibited potent scavenging activity for superoxide radicals in a concentration dependent manner with almost similar EC_50_ values of 52 and 55 *μ*g/mL, respectively (Table 2). Root extracts (aqueous and alcoholic) also exhibited good scavenging activity. Ethanolic extract of root is more potent in activity than its aqueous extract with EC_50_ values of 201 *μ*g/mL which is less than EC_50_ of latter (391 *μ*g/mL) (Table 2).

### 3.3. Lipid Peroxidation Assay

Both extracts of *P. fraternus *inhibited lipid peroxidation, induced by ferrous sulfate in egg-yolk homogenates in a concentration dependent manner. Interestingly, there was no significant difference in the EC_50_ values of both ethanolic and aqueous extract of aerial part which is about 1522 *μ*g/mL and 1533 *μ*g/mL, respectively (Table 3). Aqueous extracts of root have less lipid peroxidation inhibitory activity than ethanolic extract with EC_50_ value of 3547 *μ*g/mL and 1957 *μ*g/mL, respectively (Table 3).

### 3.4. Reducing Power Assay

**Figure 1:**
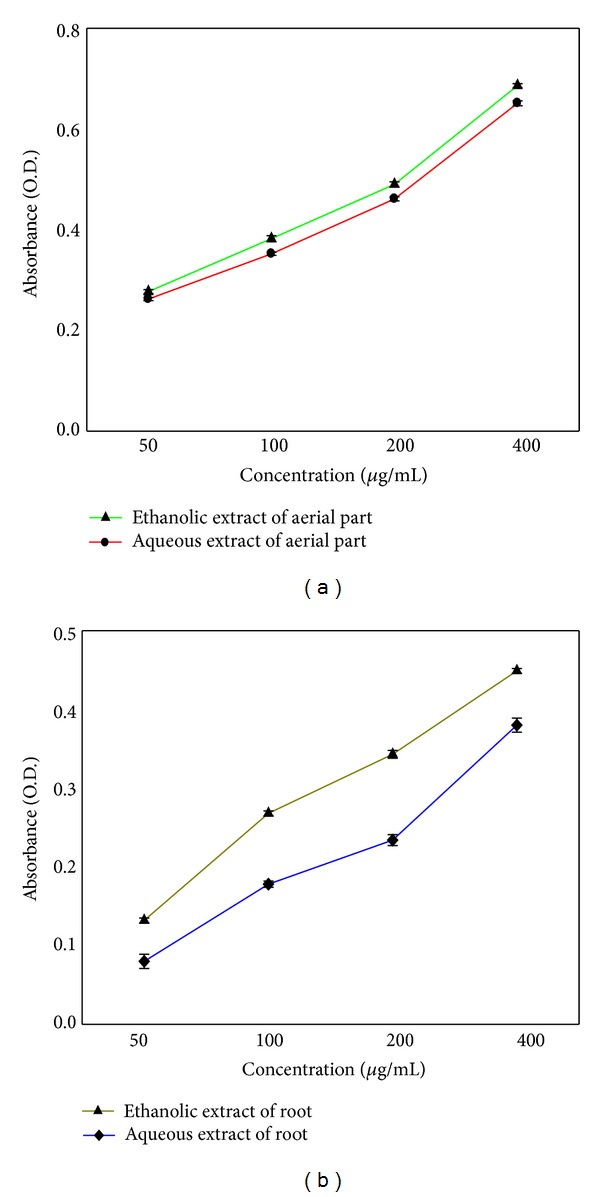
Reducing power of extracts of *P. fraternus*. (a) Ethanolic and aqueous extracts of aerial part. (b) Ethanolic and aqueous extracts of root. Results are mean ± SE of three independent experiments.

The reducing power of both aqueous and ethanolic extracts increased in concentration dependant manner. Ethanolic extracts of both aerial part and root have greater reducing power in comparison to their aqueous extract. There is no significant difference in reducing power of both extracts of aerial parts while aqueous extracts of root have lesser reducing capacity in comparison to its ethanolic extract (Figures 1(a) and 1(b)).

### 3.5. Total Phenolic Content

**Figure 2:**
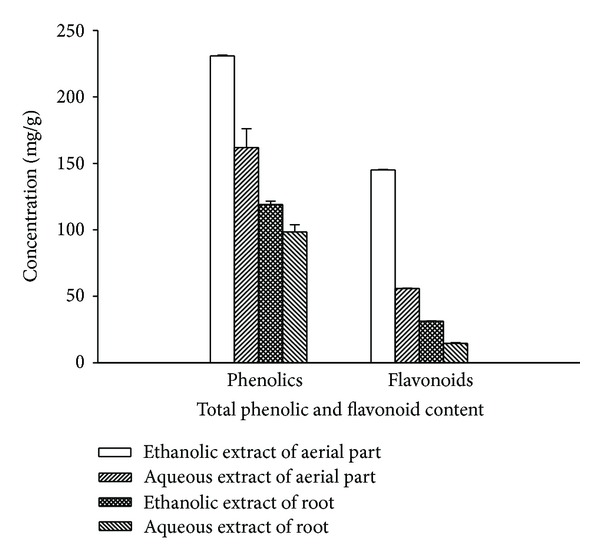
Total phenolic and flavonoid content in *P. fraternus* aerial part and root.

TP content, as determined by Folin-Ciocalteu's method, is reported as GAE by reference to standard curve (*y* = 0.026*x*, *R* ^2^ = 0.993). Phenolic content is higher in the ethanolic extract of aerial part than its aqueous extract which is about 230.85 ± 0.59 mg/g and 161.92 ± 14.12 mg/g GAE, respectively. Similarly, ethanolic extract of root is greater in phenolic content (118.94 ± 2.69 mg/g GAE) than aqueous extract (98.37 ± 5.47 mg/g GAE) (Figure 2).

### 3.6. Total Flavonoid Content

Total flavonoid content was determined by AlCl_3_ colorimetric method and reported as RE by reference to standard curve (*y* = 0.009*x*, *R* ^2^ = 0.998). Total flavonoids are higher in ethanolic extract of both plant parts than aqueous extract. Ethanolic extracts of aerial part have flavonoid content of 145.03 ± 0.26 mg/g RE while aqueous extracts have 55.83 ± 0.31 mg/g RE. Total flavonoid content is about 31.23 ± 0.14 mg/g and 14.60 ± 0.52 mg/g RE in ethanolic and aqueous part of root, respectively (Figure 2).

### 3.7. Correlation between Total Antioxidant Activity and Polyphenolic Contents (TP&TF)

**Figure 3:**
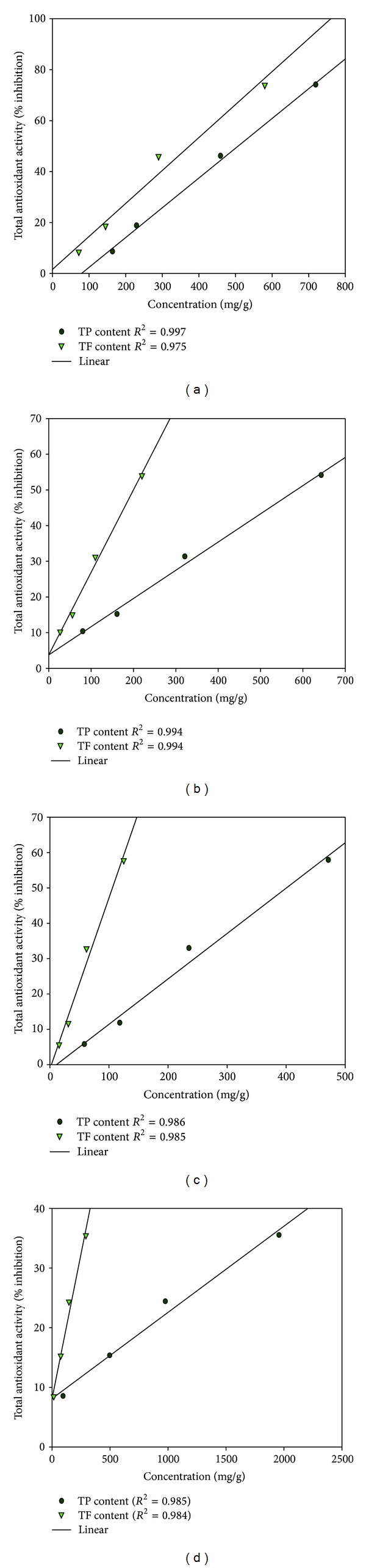
Correlation between total antioxidant activity and polyphenolic (TP&TF) contents of different plant parts of *P. fraternus*. (a) Ethanolic extract of aerial Part. (b) Aqueous extract of aerial part. (c) Ethanolic extract of root. (d) Aqueous extract of root.

Very close correlation was obtained between total antioxidant activity and polyphenolic contents (TP&TF) of various extracts which is clear from their correlation coefficient (*R* ^2^) values (Figure 3). A very high linear correlation was established between antioxidant activities and TP and TF content of different extracts (*R* ^2^ values ≥ 0.975).

## 4. Discussion 

In present study two different solvents, namely, water and ethanol, were used for extraction of dried aerial parts and roots of *P. fraternus*. The DPPH method is an easy, rapid, stable, and sensitive way to determine the antioxidant activity of a specific compound or plant extracts [21]. In this assay, DPPH free radical accepts hydrogen and gets reduced by an antioxidant. Both aqueous and ethanolic extract of aerial parts and roots have shown steady increase in percentage inhibition by DPPH radicals with increasing concentration (Table 1). Ethanolic extract of both plant parts have greater inhibitory activity than aqueous extract. The radical scavenging activity of aqueous extract of root was much lower than the ethanol extract suggesting that the antioxidants in the aqueous extract of root are weak radical-scavengers and required extremely high concentration to have a significant effect.

Superoxide radical is known to be a very harmful species to cellular components because they are precursor of more reactive oxygen species [22]. The superoxide radical is known to be produced in vivo and can result in the formation of H_2_O_2_ via dismutation reaction. Moreover, the conversion of superoxide and H_2_O_2_ into more reactive species, for example, the hydroxyl radical (OH^•^), has been thought to be one of the unfavourable effects caused by superoxide radicals [23]. In our study, extracts of both plant parts are found to be an efficient scavenger of superoxide radical generated in riboflavin-NBT-light system in vitro and their activity increased with increase in concentration of extract (Table 2). There is no significant difference in scavenging activity of both aqueous and ethanolic extract of aerial part (Table 2). However, ethanolic extracts of root have greater scavenging activity than its aqueous extract which might be due to weak scavenging activity of compounds in aqueous extract. Lipid peroxidation is a free radical-initiated oxidative chain reaction in which one lipid molecule after another becomes oxidized to the maximum possible extent or so as to form lipid peroxide. Normally, this chain reaction is terminated when the substrate is depleted. Other condition includes the combination of two radicals to form nonradical product or reaction with antioxidants, which provide easily donatable hydrogen for abstraction by peroxyl radicals. This assay is done by either enzymatic (Fe/NADPH) or nonenzymatic (Fe/ascorbic acid) method. Since we have used egg-yolk as a substrate it could be suggested that *P. fraternus* is active against nonenzymatic oxidation. Pandey et al. [24] have also reported non enzymatic method of lipid peroxidation by using different fractions of tubers from *Pueraria tuberosa* Linn. In our study both extract of aerial parts causes similar inhibition of lipid peroxidation. However, ethanolic extracts of root have greater inhibitory activity than aqueous extract. In reducing power assay, the yellow colour of the test solution changes to various shades of green and blue, depending on the reducing power of extract. The presence of reducers in sample causes the reduction of the Fe^3+^/ferricyanide complex to the Fe^2+^ form. Thus, the higher the reducer concentration, the higher the amount of Fe^2+^ which becomes evident with high absorbance of sample. These reducers show their antioxidant action by breaking the free radical chain by donating a hydrogen atom [25] and also react with certain precursors of peroxide, which in turn prevents peroxide formation. Some earlier workers [26] have observed a direct correlation between antioxidant activity and reducing power of certain plant extracts. In the present study also, reducing power of all extracts increases with increase in concentration which is in close correlation with its observed antioxidant activity. Thus, these reducers must be responsible for antioxidant property of extracts.

Phenolic compounds are secondary metabolites of plants and can act as antioxidants by many potential pathways such as free radical scavenging, oxygen radical absorbance, and chelation of metal ions [27]. Phenolic content is greater in ethanolic extract of both plant parts than its aqueous extract which might be responsible for high antioxidant activity and reducing power of respective extract. Some earlier workers have also found good correlation in phenolic content and antioxidant activity of plant extract [28, 29]. Flavonoids are well-known antioxidant constituents of plants and possess a broad spectrum of chemical and biological activity, including radical scavenging properties [30]. Therefore, flavonoid content of the extracts was also analyzed. There was a good correlation between antioxidant activity of plant extracts, and its TP and TF content.

## 5. Conclusions

In conclusion, extracts of *P. fraternus* have potent antioxidant activity and reducing capacity. They are rich source of polyphenols like phenolics and flavonoids. Their antioxidant activities are quite correlated with their polyphenolic contents and reducing power. Among both plant parts, namely, aerial parts and root, aerial part shows greater antioxidant property which is possibly due to its higher polyphenolic content. Higher antioxidant property of ethanolic extract than aqueous extracts might be due to greater solubility of active constituents in ethanol than in water. So, these extracts could be used as new sources of natural antioxidants for treatment of oxidative stress induced diseases. It can be supplemented in nutraceutical products as dietary supplements. It may be used as preservatives in food and cosmetics as well as to prevent the degradation of rubber and gasoline.

## Figures and Tables

**Table 1:** DPPH free radical scavenging activity of *P. fraternus* aqueous and ethanolic extracts of aerial part and root.

Aerial part			Root		
Concentration (µg/mL)	Aqueous extract	Ethanol extract	Ethanol extract	Concentration (*µ*g/mL)	Aqueous extract
	Percentage inhibition (mean ± SE)	Percentage inhibition (mean ± SE)	Percentage inhibition (mean ± SE)		Percentage inhibition (mean ± SE)
50	10.28 ± 0.72	8.47 ± 0.47	5.67 ± 0.68**	100	8.47 ± 0.28**
100	15.09 ± 0.51**	18.64 ± 0.99**	11.72 ± 1.36	500	15.29 ± 0.67**
200	31.23 ± 1.54**	45.99 ± 1.15**	32.85 ± 0.54**	1000	24.35 ± 1.11**
300	40.16 ± 1.33**	61.18 ± 1.32**	39.28 ± 1.68**	2000	35.46 ± 0.61**
400	54.04 ± 1.91**	73.96 ± 1.01**	57.77 ± 1.50**	3000	48.40 ± 0.52**
500	62.92 ± 1.89**	94.59 ± 1.10**	64.91 ± 1.33**	4000	63.86 ± 0.78**
				5000	77.98 ± 0.76**
EC_50_	360	258	337	EC_50_	3038

EC_50_ of Ascorbic acid is 88.96.

**Significant at *P *< 0.001.

**Table 2:** Superoxide radical scavenging activity of *P. fraternus* aqueous and ethanolic extracts of aerial part and root.

Aerial part			Root		
Concentration (µg/mL)	Aqueous extract	Ethanol extract	Concentration (µg/mL)	Aqueous extract	Ethanol extract
	Percentage inhibition (mean ± SE)	Percentage inhibition (mean ± SE)		Percentage inhibition (mean ± SE)	Percentage inhibition (mean ± SE)
20	1.81 ± 0.32	6.64 ± 1.53	50	14.08 ± 0.93**	7.6 ± 0.88**
40	48.04 ± 0.86**	30.06 ± 1.36**	100	17.73 ± 0.10**	29.20 ± 0.46**
60	64.51 ± 0.32**	77.32 ± 2.95**	200	24.27 ± 1.21**	62.14 ± 1.08**
80	80.32 ± 0.46**	88.81 ± 0.56**	400	49.78 ± 0.51**	88.09 ± 0.94**
100	99.98 ± 0.03**	—	600	78.02 ± 0.20**	95.55 ± 0.48**
			800	93.72 ± 0.49**	—
EC_50_	52	55	EC_50_	391	201

EC_50_ of Copper sulphate is 4.18.

**Significant at *P *< 0.001.

**Table 3:** Lipid peroxidation activity of *P. fraternus* aqueous and ethanolic extracts of aerial part and root.

Concentration (µg/mL)	Percentage inhibition (mean ± SE)			
	Aerial Part		Root	
	Aqueous extract	Ethanol extract	Aqueous extract	Ethanol extract
500	18.22 ± 1.05	20.76 ± 1.05	—	15.97 ± 0.62**
1000	42.0 ± 1.73**	47.92 ± 3.84**	9.85 ± 4.66	39.00 ± 0.41**
2000	62.34 ± 1.52**	63.22 ± 0.75**	31.16 ± 1.18**	54.83 ± 0.27**
3000	81.07 ± 0.64**	83.45 ± 0.54**	45.54 ± 0.12**	69.74 ± 0.82**
4000	93.34 ± 0.48**	96.55 ± 0.27**	57.04 ± 1.63**	83.92 ± 0.62**
6000	—	—	85.57 ± 0.30**	—
EC_50_	1533	1521	3547	1957

EC_50_ of Ascorbic acid is 643.89.

**Significant at *P* < 0.001.
